# COVID-19 Pandemic: Perspective From Italian Pediatric Emergency Physicians

**DOI:** 10.1017/dmp.2020.198

**Published:** 2020-06-22

**Authors:** Alice Monzani, Luca Ragazzoni, Francesco Della Corte, Ivana Rabbone, Jeffrey M. Franc

**Affiliations:** Division of Pediatrics, Department of Health Sciences, Università del Piemonte Orientale, Novara, Italy; CRIMEDIM - Research Center in Emergency and Disaster Medicine, Università del Piemonte Orientale, Novara, Italy

**Keywords:** COVID-19, emergency department, pediatrics

## Abstract

**Objectives:**

To document the lived experience of Italian pediatric emergency physicians during the coronavirus disease 2019 (COVID-19) pandemic.

**Methods:**

We developed a structured interview to collect the lived experience of the staff of the pediatric emergency department (PED) of a tertiary referral university hospital in Northern Italy. The open-ended questions were draft according to the suggestions of Canadian colleagues and administered by 1 interviewer, who was part of the PED staff, at the end of March 2020. All the PED staff was interviewed, on a voluntary basis, using purposive sampling.

**Results:**

Most respondents declared to be afraid of becoming infected and of infecting their families. The number of patients seen in the PED has decreased, and the cases tend to be more severe. A shift in the clinical approach to the ill child has occurred, the physical examination is problem-oriented, aiming to avoid un-necessary maneuvers and to minimize the number of practitioners involved. The most challenging aspects reported are: (1) performing a physical examination in personal protective equipment (PPE), (2) being updated with rapidly evolving guidelines, and (3) staying focused on the possible COVID-19 clinical presentation without failing in differential diagnosis.

**Conclusions:**

During the COVID-19 pandemic, it seems that pediatric emergency physicians are radically changing their clinical practice, aiming at prioritizing essential interventions and maneuvers and self-protection.

The coronavirus disease 2019 (COVID-19) pandemic is now threatening several health systems around the world. In Northern Italy, being 1 of the most affected areas so far, emergency departments are still struggling to deal with mass influxes of patients.^1^ Even though severe COVID-19 in children is rare and the number of confirmed cases in the pediatric population is significantly smaller compared with adults, pediatric emergency departments (PEDs) remain a crucial hospital service during such a public health crisis.^2,3^ Pediatricians are required to apply infection prevention and control (IPC) measures and operational public health skills, also encompassing direct patient care for suspected or confirmed patients with COVID-19 and the proper application of personal protective equipment (PPE). Unfortunately, these competencies are beyond the experience and knowledge base of many health-care workers who are not familiar with public health emergencies arising from infectious disease outbreaks. Clinicians may feel uneasy about treating an infectious disease for which there is no vaccine, for which there is no specific therapy, and which is highly transmissible. In Italy, the risk for COVID-19 secondary infections among health-care workers is extremely high, which highlights the probably inadequate IPC practices and protections in place in many hospitals and primary health-care services.^4^

In this scenario, numerous research studies and practical and clinical guidelines have been published by the World Health Organization and other academic institutions to increase the safety among practitioners and to enhance the quality of care. However, to the best of our knowledge, few scientists have studied the current pandemic from the perception of health-care workers providing pediatric care on the frontline. Fear and challenges to respond to infectious emergencies, including pandemic influenza, are already well documented.^5^ Nevertheless, little is known about the health-care workers’ perspective on COVID-19 pandemic, especially from pediatric emergency physicians.

The objective of this study was to document the lived experience of pediatric health-care providers in Italy during the initial phase of the 2020 COVID-19 pandemic. The main areas of investigation were the variation in their hospital daily activities and the challenges encountered during direct patient care.

## METHODS

We developed a structured interview to collect the lived experience of pediatric health-care providers of the Maggiore della Carità University Hospital (Novara, Italy), the second largest third level referral hospital of the Piedmont Region, one of the most COVID-19 affected regions in northwest Italy. The open-ended questions, draft according to the suggestions and interests of Canadian pediatric emergency physicians, were collected at the end of March 2020, when 101,739 confirmed cases were reported in Italy, with 11,591 deaths, whereas Canada was not already critically involved in the COVID-19 outbreak. Canadian pediatric emergency physicians were interested in knowing the real-life experience of their Italian counterparts. One of the authors (J.M.F.) collected by email the questions of his Canadian colleagues. These open-ended questions represented the basis for the development of the interview. Questions with similar content were merged to produce the final open-ended questions included in the interview. The PED staff of the Maggiore della Carità University Hospital agreed to partake in the interview process. The questions were administered by 1 interviewer (A.M.) who was part of the PED staff. All the staff of the PED were interviewed, either by face-to-face or phone interviews, on a voluntary basis. We used a purposive sampling, including the complete target population. The face-to-face interviews took place in the hospital, whereas the phone interview took place when the respondents were either in the hospital or at home. The day, time, and location of the interview were scheduled according to the respondents’ availability and convenience, within a 1-wk frame, given the timeliness of the study. The comments and perceptions were collected and summarized to answer the open-ended questions. The written transcripts were read several times to obtain an overall feeling for them. Relevant phrases or sentences that pertained directly to the lived experience were identified. Integrated meanings were then formulated from the relevant statements and phrases, to provide an exhaustive summary of single perceptions, as detailed by Creswell and Poth.^6^ The participation in the interview was considered as the consent to participate in this report. Confidentiality of information was ensured, and no financial incentive to participate in the study was offered. Because no identifying personal data are reported and the responses to the interview are presented in aggregate form, the study was deemed exempt from the requirement for formal approval by the local Ethics Committee.

## RESULTS

All the 13 physicians working in our PED (M:F = 11:2; aged 33-60 years) took part to the interviews, which lasted approximately 30 min.

### Theme 1. Are You Afraid of Becoming Infected or Infecting Your Family?

Most of the respondents said that they are afraid of becoming infected, mainly because the use of PPE is reserved for patients with fever and/or respiratory symptoms, but it is known that also asymptomatic subjects may spread the virus. Therefore, the only way to protect yourself would be if patients wore surgical masks, but it is not so feasible for younger children or, even more, for neonates. All the respondents are afraid of infecting their families so that most of them have changed their lifestyle. Some pediatricians are still living at home with their families, but sleeping in a separate bed or bedroom, keeping distances from their family members, and, in some cases, wearing surgical masks also at home. A few other colleagues decided to leave their families and went to stay in hotels or empty houses to minimize the interactions with their cohabitants and the risk of infecting them.

### Theme 2. Has the Number and/or Type of Patients Seen in Your Emergency Department Changed During the COVID-19 Pandemic?

The number of patients seen in our PED has dramatically decreased (approximately 70-80% reduction) during the COVID-19 pandemic. All the respondents thought this could be due both to the restriction measures that came into force on March 9, and to the fear of the patients to be infected coming to the hospital. As a result, also the type of patients admitted has changed. The less severe codes (white and green codes) are decreased, alongside an increase in more severe ones was seen, suggesting that patients with minor and milder conditions tend to avoid seeking the PED. A considerable increase in traumatic injuries was noticed, perhaps linked to the motor hyperactivity often experienced by children under restrictive measures, forced to play in the limited space of their houses. Moreover, surely, there are children referring to the PED for suspected COVID-19, presenting with fever, cough, sore throat, abdominal pain/diarrhea, skin lesions, and often with family members with COVID-19. Nonetheless, as it is known that COVID-19 in children may often be asymptomatic, it is important to keep a high level of suspicion with all the children admitted to the ED, also for conditions not directly linked to COVID-19 (ie, injuries, burns, foreign body ingestions…).

### Theme 3. How Has Your Practice/Attitude Changed During the Physical Examination of the Patients in the PED?

The respondents’ attitude during physical examination has considerably changed during the COVID-19 pandemic. Certainly, wearing PPE represents a substantial change and makes it difficult to perform some maneuvers, for example, the face shield prevents an accurate otoscopy. More broadly, a radical shift in the approach to the ill child has occurred. Pediatricians are used to meticulous head-to-toe physical examinations. In this unique situation, the focus has changed. Physicians must protect themselves as a priority, so that the physical examination is problem-oriented and aimed at avoiding unnecessary maneuvers, also in a PPE-sparing perspective. For example, if a child is referred for a foot injury, a throat examination is not performed, so that the physician would not require to wear complete equipment, such as goggles or face shields.

Another substantial change is the attitude toward fever. If pediatricians used to consider as a noteworthy fever only a body temperature higher than 38-38.5°C, now even a rising of a few degrees in body temperature requires attention. It must be highlighted that in the lockdown period, when children are not attending schools, common community-acquired diseases are extremely uncommon so that the detection of fever should suggest the possibility of COVID-19. In this case, the patient has to be treated as potentially infected, following a specific intra-hospital patient flow, with early isolation, and should be tested for the presence of severe acute respiratory syndrome coronavirus 2 (SARS-Cov-2) RNA.

### Theme 4. Are You Limiting the Number of Family Members That Are Allowed in the Examination Room?

All the respondents have changed their attitude and now only one parent is allowed in the examination room, aiming at limiting the contacts for health-care practitioners, even if it could be less reassuring for the youngest patients.

### Theme 5. How Do You Think That Patients and Families Feel About Seeing You in PPE?

It is a completely new situation. Some of the respondents pointed out that children could be scared by such unusual stuff, but one can pretend to be an astronaut to try to add a little magic to a potentially frightening setting. There was wide agreement about the fact that most of the parents look comforted by the use of PPE and are cooperative in reassuring their kids.

### Theme 6. Do You Think You Can Miss Important Diagnoses Because of Concerns About PPE and COVID-19?

This seems to be one of the main concerns of the respondents. On the one hand, they are worried about the possibility that the focus on COVID-19 may lead to fixation errors, detracting from other differential diagnoses that should be considered in the presence of fever and respiratory symptoms. On the other hand, there is also concern that seriously ill children could delay the admission to the PED for fear of becoming infected in the hospital.

### Theme 7. Has Your Behavior With Your Colleagues/Collaborators Changed?

Most of the respondents emphasized that the interactions with the colleagues are limited to the strict minimum necessary, keeping the right distances also when discussing patients, and minimizing the number of practitioners involved in the same procedure.

### Theme 8. What Is the Most Challenging Aspect of the Current Situation?

From the interviews, multiple challenging aspects in the management of the COVID-19 pandemic in a PED were collected. Physicians have to face with the new dimension of performing a physical examination in PPE; they have to be trained and skilled in proper donning/doffing procedures; they have to keep constantly updated with rapidly evolving guidelines and protocols; they have to be focused on the possible SARS-CoV-2 infection, but, at the same time, they should not forget differential diagnoses.

## DISCUSSION

Exploring for the first time the perception of pediatric emergency physicians in one of the most affected countries, this study represents a unique attempt to provide hints to those pediatricians not already critically involved in facing the COVID-19 pandemic across the world. Similar insights from Chinese colleagues, initially involved in COVID-19 outbreak management, would have been extremely useful at the very onset of the pandemic, to be at least in part prepared in advance to what would expect pediatricians around the world. One of the key points is that the clinical practice changed, and individual attitude was modified, aiming at prioritizing essential interventions and maneuvers; at the expense of routine but nonvital procedures. Health-care practitioners should be able to keep updated with the rapidly evolving knowledge and guidelines, and to comply with a way of acting that would probably be new and unfamiliar for most of them, encompassing not only disease epidemiology, IPC protocols, and application of PPE, but also basic principles of disaster medicine, ie, surge capacity and scarce resource allocation, triage, and ethical dilemmas of rationing medical care.

Another paramount notion is the absolute priority of the safety of the health-care practitioner, not only in an individual perspective but also in the community interest. Indeed, physicians not protecting themselves from the infection would turn out to be harmful for the global health-care system, both because they could not be able to work if they get sick and, probably even more, because they may spread the infection if they are asymptomatic and keep having contacts with patients and colleagues. The crucial role of proper PPE use is actually highlighted by ad-hoc COVID-19 pediatric resuscitation algorithms, released by the American Heart Association, having the donning of PPE as the first point.^7^

In this perspective, pediatricians should consider each patient as a potentially COVID-19-positive patient but should be able not to fixate only on COVID-19 disease, always bearing in mind more common differential diagnoses. Underneath the same presenting symptoms of COVID-19, there is a wide range of pathological conditions that need to be recognized and treated as well.

Finally, in countries facing with COVID-19 pandemic, it is likely that a substantial reduction in the amount of access to the PED will be seen, similarly to our experience and to that reported in other Italian centers,^8^ as a result of both the reticence on the part of the parents and caregivers to risk exposure to SARS-CoV-2 in a hospital setting, and the lower rates of acute infections in this period. This could result in a detrimental delayed provision of care for critically ill children. Lazzerini et al. reported 6 cases admitted to intensive care unit and 4 deaths related to delayed access to care, in a small series of 12 reports.^8^ On the other hand, the dramatic reduction in PED visits should provide food-for-thought on the actual appropriateness of a large part of the ordinary use of the PED to manage not urgent/emergent needs. It is advisable that it become a matter of debate for health-care policies.

This work is not intended to recommend a code of conduct but just to apprise other pediatric health-care providers not already deeply involved in the management of COVID-19 pandemic of what they would probably face in the next future.

We think that the main strength of this work is sharing real-life experiences of front-line practitioners with their peers in countries not already deeply facing the pandemic, offering insights to be to some extent prepared in advance to a completely new and unknown situation.

However, some limitations should be pointed out. First, the crisis pediatricians were facing in Italy at that time prevented us from a strict methodological structure and sample size, thus, not allowing us to provide evidence-based data. Therefore, it was just for documentation. Second, the interviews were not audio-recorded, preventing us to report unedited narratives. Finally, the fact that the interviewer was also a physician who worked with the target population in the same hospital may represent both an advantage and a disadvantage. On the one hand, it could be an advantage because it would allow a real empathetic listening of the colleagues’ opinions during the interview and the possibility to easily keep in touch with them regardless of the complexity of the COVID-19 period. On the other hand, it could represent a disadvantage possibly preventing an objective and unemotional interpretation of the responses, but this limitation was overcome by the other authors’ contribution in reviewing and integrating the written transcripts of the interviews.

In conclusion, pediatricians directly experiencing the effects of COVID-19 pandemic in their PED have radically changed their clinical practice, aiming at prioritizing essential interventions and maneuvers and self-protection. Their point of view could help their peers around the world to be prepared to face this unique situation, just trying to answer what they are wondering.

## References

[1] Paganini M, Conti A, Weinstein E et al. Translating COVID-19 pandemic surge theory to practice in the emergency department: how to expand structure. Disaster Med Public Health Prep. 2020:1-10. doi: 10.1017/dmp.2020.57 PMC7156581 32216865

[2] Ludvigsson JF. Systematic review of COVID-19 in children shows milder cases and a better prognosis than adults. Acta Paediatr. 2020;109(6):1088-1095. doi: 10.1111/apa.15270 32202343 PMC7228328

[3] Rasmussen SA, Thompson LA. Coronavirus disease 2019 and children: what pediatric health care clinicians need to know. JAMA Pediatr. 2020. doi: 10.1001/jamapediatrics.2020.1224 32242896

[4] Anelli F, Leoni G, Monaco R, et al. Italian doctors call for protecting healthcare workers and boosting community surveillance during covid-19 outbreak. BMJ. 2020;368:m1254. doi: 10.1136/bmj.m1254 32217525

[5] Errett NA, Barnett DJ, Thompson CB, et al. Assessment of medical reserve corps volunteers’ emergency response willingness using a threat- and efficacy-based model. Biosecur Bioterror. 2013;11(1):29-40. doi: 10.1089/bsp.2012.0047 PMC3612278 23477632

[6] Creswell JW, Poth CN. Qualitative Inquiry and Research Design: Choosing Among Five Approaches. 4th ed Thousand Oaks, CA: Sage Publications Inc; 2018.

[7] American Heart Association. Coronavirus (COVID-19): resources for CPR training & resuscitation - resources for healthcare providers. New AHA Scientific Statement - April 9, 2020 https://cpr.heart.org/en/resources/coronavirus-covid19-resources-for-cpr-training. Accessed April 20, 2020.

[8] Lazzerini M, Barbi E, Apicella A, et al. Delayed access or provision of care in Italy resulting from fear of COVID-19. Lancet Child Adolesc Health. 2020;4(5):e10-e11. doi: 10.1016/S2352-4642(20)30108-5 32278365 PMC7146704

